# Case Report: The application of associating liver partition and portal vein ligation for staged hepatectomy in patients with hepatitis b virus-related hepatocellular carcinoma after undergoing treatment with an immune checkpoint inhibitor: a report of two cases

**DOI:** 10.3389/fimmu.2023.1159885

**Published:** 2023-05-09

**Authors:** Cong Ning, Guanmo Liu, Junwei Zhang, Xiaobo Yang, Yiyao Xu, Haitao Zhao

**Affiliations:** ^1^ Department of Liver Surgery, Peking Union Medical College Hospital, Chinese Academy of Medical Sciences and Peking Union Medical College (CAMS and PUMC), Beijing, China; ^2^ Department of Breast Surgery, Peking Union Medical College Hospital, Chinese Academy of Medical Sciences and Peking Union Medical College (CAMS and PUMC), Beijing, China

**Keywords:** unresectable hepatocellular carcinoma, immunotherapy, associating liver partition and portal vein ligation for staged hepatectomy, future liver remnant, immune checkpoint inhibitors

## Abstract

Hepatocellular carcinoma (HCC) is often diagnosed at an unresectable stage without opportunities for curative therapy. Future liver remnant (FLR) insufficiency limits the range of patients who can undergo radical resection. Associating liver partition and portal vein ligation for staged hepatectomy (ALPPS) can ultimately achieve short-term hypertrophy of the FLR in patients with viral hepatitis-related fibrosis/cirrhosis and R0 resection. However, the influence of immune checkpoint inhibitors (ICIs) on liver regeneration remains unknown. We report two patients diagnosed with Barcelona Clinic Liver Cancer (BCLC)-B stage hepatitis B virus (HBV)-related HCC who underwent pioneering ALPPS after immunotherapy without posthepatectomy liver failure (PHLF). ALPPS has been shown to be safe and feasible in patients with HCC who underwent immunotherapy previously for the first time and might provide an alternative salvage option for future conversion therapy of HCC.

## Introduction

Hepatocellular carcinoma (HCC) is one of the most common malignancies in the world, ranking sixth in morbidity and fourth in cancer-related deaths (1). Majority of patients are diagnosed at an advanced stage without the opportunity to receive curative therapies including ablation and surgery (2). The volume of the future liver remnant (FLR) acts as an important limiting factor in the determination of tumour resectability (3). Approximately 80% of patients with HCC have a background of chronic hepatitis B virus (HBV) or hepatitis C virus (HCV) infection and liver cirrhosis, and severity of these conditions is negatively correlated with the rate of hypertrophy of the FLR (3). Posthepatectomy liver failure (PHLF) caused by FLR insufficiency eliminates of the hope of radical resection (4).

Associating liver partition and portal vein ligation for staged hepatectomy (ALPPS) can achieve rapid FLR growth in two weeks with a low risk of tumour progression during therapy (5). ALPPS has been shown to be safe and feasible in selected patients with viral hepatitis-related HCC (6).

Immune checkpoint inhibitors (ICIs) can restore the antitumor function of effector T cells through the inhibition of immune checkpoint molecules such as programmed cell death protein 1 (PD-1) and cytotoxic T lymphocyte-associated antigen-4 (CTLA-4), which are revolutionizing the management of HCC (7–9). ICIs also affect other PD-1-expressed cells such as B cells, macrophages, and natural killer T (NKT) cells, and inflammatory processes in tumor microenvironment (10–12). It’s worth noting that liver regeneration and hepatocarcinogenesis involve multiple common events and processes (13). However, the exact association between ICIs and liver hypertrophy after hepatectomy remains unclear.

In the era of immunotherapy for solid tumors, the application of ALPPS in HCC patients with fibrosis/cirrhosis receiving ICIs has not been reported previously. Here, we report two cases of Barcelona Clinic Liver Cancer (BCLC)-B stage HCC patients who received combination therapy followed by pioneering ALPPS and completed conversion therapy successfully without serious perioperative complications.

## Case presentation

### Case 1

**Figure 1:**
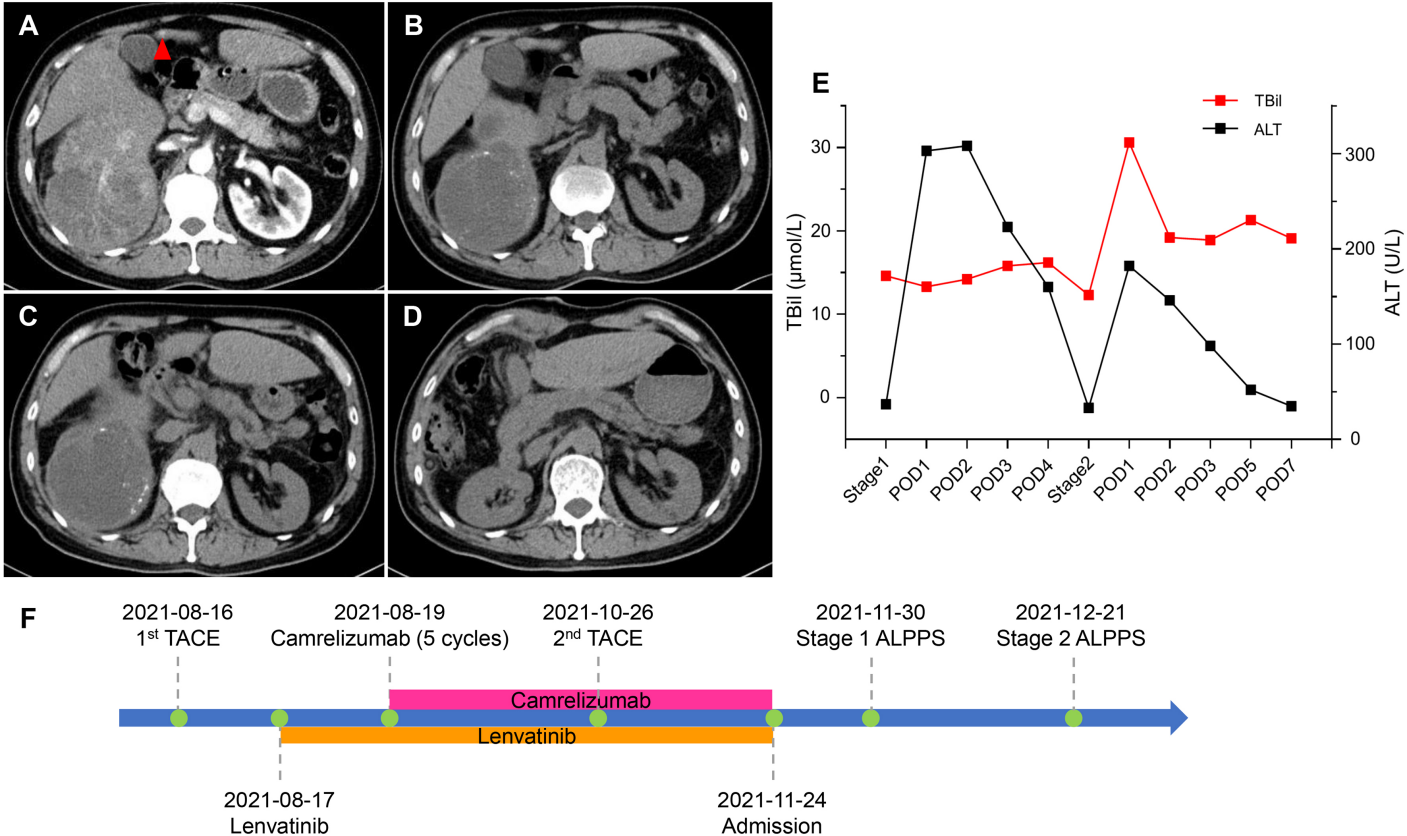
CT scans and changes in liver function of 1st patient. **(A)** Arterial phase of CT before stereotactic therapy. A lesion (red triangle) measuring 1.5 cm × 1.0 cm in liver segment 4b. **(B)** Plain scan before stage 1 ALPPS. **(C)** Plain scan before stage 2 ALPPS. **(D)** Plain scan 7 days after stage 2 ALPPS. **(E)** Changes in liver function during ALPPS. After stage 1, TBil remained a stable level, whereas ALT transiently rose but quickly declined. After stage 2, TBil showed no inordinate increases and quickly returned to normal, and ALT showed no significant upturns. **(F)** Timeline of treatment process. ALPPS, associating liver partition and portal vein ligation for staged hepatectomy; ALT, alanine aminotransferase; CT, computer tomography; POD, postoperative day; TACE, transarterial chemoembolization; TBil, total bilirubin.

A 54-year-old male presented to our hospital because of upper abdominal pain. The patient weighed 65 kg, his height was 167 cm, and the standard liver volume (SLV) was 1231 mL, which was calculated based on the Urata formula (14). Laboratory examination (**Table 1**) showed alpha fetoprotein (AFP) >60500 ng/mL, total bilirubin (TBil) 20.4 μmol/L, direct bilirubin (DBil) 7.1 μmol/L, alanine transaminase (ALT) 69 U/L, and hepatitis B surface antigen (HBsAg) (+). Abdominal enhanced computed tomography (CT) indicated liver cirrhosis, a 14.9 cm × 11.8 cm mass located in the right liver and a 1.5 cm × 1.0 cm mass located in the left liver both with heterogeneous rapid enhancement of the mass in the arterial phase (**Figure 1A**). The patient was diagnosed with chronic hepatitis B from childhood and had regular antiviral therapy while finding the masses in the liver. The clinical diagnosis was HCC with BCLC-B stage. According to the BCLC strategy, transarterial chemoembolization (TACE) and a combination of the targeted drug (lenvatinib, 8 mg once a day) and ICIs (camrelizumab, 200 mg every three weeks) were performed (**Figure 1F**). After two courses of TACE and five cycles of immunotherapy, abdominal enhanced CT showed that the range of enhancement was significantly smaller than before. Although the diameter of the lesion was slightly smaller, the lesion reached partial response (PR; i.e., >30% decrease in the sum of diameters of viable (enhancing) target lesions) according to modified Response Evaluation Criteria Solid Tumours (mRECIST) (15). Laboratory examination before the surgery showed AFP 167.0 ng/mL, TBil 14.6 μmol/L, DBil 5.9 μmol/L, aspartic transaminase (AST) 41 U/L, and ALT 37 U/L. The volume of FLR was 475 mL, as calculated by abdominal CT (**Figure 1B**). An insufficient FLR could be linked to a high risk of postoperative liver failure after first-stage resection. After having obtained the consent of the patient and his family, the first stage of ALPPS and resection of the left lesion by laparoscopy was performed three months after the first course of TACE. A postoperative liver function test (LFT) indicated TBil 13.3 μmol/L, DBil 5.5 μmol/L, and ALT 303 U/L. Liver function improved after liver protection therapy. Twenty-one days after the first-stage surgery, abdominal CT indicated that the FLR increased to 576 mL (**Figure 1C**), and the rate of FLR/SLV was 46.8%, which could meet the needs of the body. The second stage of ALPPS was completed, and the TBil, DBil and ALT were 30.6 μmol/L, 13.3 μmol/L and 182 U/L, respectively, on the first day after surgery. Changes in liver function are shown in **Figure 1E**. The FLR was 708 mL (**Figure 1D**), and the patient was discharged 7 days after second-stage ALPPS. A pathology report demonstrated a mass in the left liver with moderately differentiated HCC and a mass in the right liver with extensive haemorrhage and necrosis and no clear residual tumour cells, which reached pathological complete response (pCR). The systemic therapy was restored two months after ALPPS with the same regimen as before in the follow-up clinic. The follow-up examination showed no recurrence on imaging and sufficient FLR on that day.

**Table 1:** Laboratory examination of patient 1.

Patient 1	Baseline	Stage 1	POD 1	POD 2	POD 3	POD 4	Stage 2	POD 1	POD 2	POD 3	POD 5	POD 7
ALT (U/L)	69	37	303	308	223	160	33	182	146	98	52	35
TBil (μmol/L)	20.4	14.6	13.3	14.2	15.8	16.2	12.3	30.6	19.2	18.9	21.3	19.1
DBil (μmol/L)	7.1	5.9	5.5	6.1	7.2	7.0	5.2	13.3	8.8	9.0	11.2	9.7
AST (U/L)	111	41					33					
ALP (U/L)	130	97					112					
γ-GGT (U/L)	129	37					39					
AFP (ng/mL)	>60500	167					72					

Baseline, the examination before commencement of stereotactic therapy; Stage 1, the latest examination before the first stage of ALPPS; POD 1-4, the examination on the corresponding day after the first stage of ALPPS; Stage 2, the latest examination before the second stage of ALPPS; POD 1, 2, 3, 5, and 7, the examination on the corresponding day after the second stage of ALPPS.

AFP, alpha fetoprotein; ALP, alkaline phosphatase; ALPPS, associating liver partition and portal vein ligation for staged hepatectomy; ALT, alanine aminotransferase; AST, aspartic transaminase; DBil, direct bilirubin; POD, postoperative day; TBil, total bilirubin; γ-GGT, γ gamma-glutamyl transpeptidase.

### Case 2

**Figure 2:**
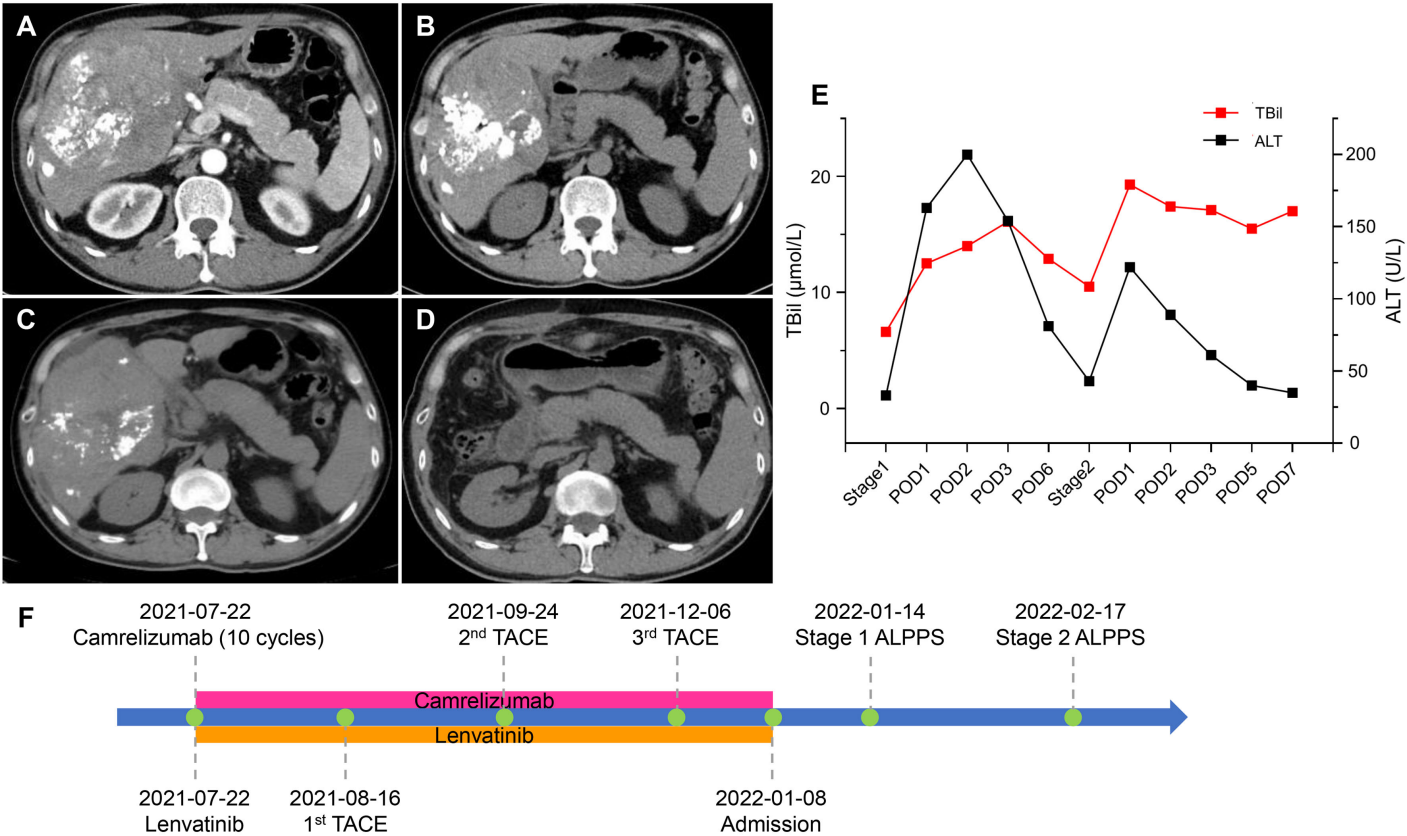
CT scans and changes in liver function of 2nd patient. **(A)** Arterial phase of CT after two courses of TACE. **(B)** Plain scan before stage 1 ALPPS. **(C)** Plain scan before stage 2 ALPPS. **(D)** Plain scan one month after stage 2 ALPPS. **(E)** Changes in liver function during ALPPS. After stage 1, TBil escalated within the normal level, whereas ALT transiently rose but quickly declined. After stage 2, TBil showed no inordinate increases, and ALT showed no significant upturns. **(F)** Timeline of treatment process. ALPPS, associating liver partition and portal vein ligation for staged hepatectomy; ALT, alanine aminotransferase; CT, computer tomography; POD, postoperative day; TACE, transarterial chemoembolization; TBil, total bilirubin.

A 48-year-old male was admitted to our hospital due to a palpable mass in the right upper abdomen. The patient weighed 60 kg with a height of 178 cm, and the SLV was 1217 mL. Laboratory examination (**Table 2**) revealed TBil 12.2 μmol/L, DBil 5.8 μmol/L, alkaline phosphatase (ALP) 1016 U/L, γ gamma-glutamyl transpeptidase (γ-GGT) 671 U/L, HBV-DNA 735780 IU/L, and HBsAg (+). AFP was at the normal level. Abdominal enhanced CT showed a 16.2 cm × 11.2 cm mass situated in the right liver with inhomogeneous enhancement of the mass in the arterial phase and multiple intrahepatic metastatic lesions (**Figure 2A**). In addition, the patient was administered interferon therapy when diagnosed with HBV. According to the BCLC-B stage, stereotactic therapy for HCC was performed (i.e., TACE combined with 8 mg lenvatinib once a day and 200 mg camrelizumab every three weeks, **Figure 2F**). Abdominal CT showed that the diameter of the lesion was reduced from 16.2 cm to 9.7 cm (**Figure 2B**) after three courses of TACE and ten cycles of camrelizumab. LFT was almost normal, except ALP 261 U/L and γ-GGT 120 U/L. The first stage of ALPPS was performed with laparoscopy after obtaining the consent of the patient and his family. LFT suggested TBil 12.5 μmol/L, DBil 5.6 μmol/L, and ALT 163 U/L on the first day after surgery. One month after the first-stage surgery, the volume of FLR was 473 mL, which was more hypertrophic than the baseline volume of FLR (356 mL) (**Figure 2C**). The rate of FLR/SLV was 38.9%, and the patient received right hepatectomy plus right caudate-leaf resection by laparotomy. Changes in liver function are shown in **Figure 2E**. The pathology report suggested moderately differentiated hepatocellular carcinoma with satellite nodules. Systemic therapy was restored one week after second-stage ALPPS. Re-examination of CT one month after second-stage ALPPS indicated sufficient FLR (**Figure 2D**).

**Table 2:** Laboratory examination of patient 2.

Patient 2	Baseline	Stage 1	POD 1	POD 2	POD 3	POD 6	Stage 2	POD 1	POD 2	POD 3	POD 5	POD 7
ALT (U/L)	63	33	163	200	154	81	43	122	89	61	40	35
TBil (μmol/L)	12.2	6.6	12.5	14.0	16.1	12.9	10.5	19.3	17.4	17.1	15.5	17.0
DBil (μmol/L)	5.8	3.1	5.6	6.6	8.1	6.7	4.8	9.1	8.7	8.9	8.8	9.0
AST (U/L)	85	45					49					
ALP (U/L)	1016	261					444					
γ-GGT (U/L)	671	120					302					
AFP (ng/mL)	13.4	5.3					26					

Baseline, the examination before commencement of stereotactic therapy; Stage 1, the latest examination before the first stage of ALPPS; POD 1, 2, 3, and 6, the examination on the corresponding day after the first stage of ALPPS; Stage 2, the latest examination before the second stage of ALPPS; POD 1, 2, 3, 5, and 7, the examination on the corresponding day after the second stage of ALPPS.

AFP, alpha fetoprotein; ALP, alkaline phosphatase; ALPPS, associating liver partition and portal vein ligation for staged hepatectomy; ALT, alanine aminotransferase; AST, aspartic transaminase; DBil, direct bilirubin; POD, postoperative day; TBil, total bilirubin; γ-GGT, γ gamma-glutamyl transpeptidase.

## Discussion

ALPPS is a surgical strategy that can achieve radical resection of surgically unresectable HBV-related HCC by short-term hypertrophy of the FLR (3). However, the survival benefit of ALPPS may be limited at the beginning of the confirmed diagnosis in patients with BCLC-B stage HCC involving both left and right livers or large masses with satellite lesions (16). Currently, most guidelines recommend TACE and/or systemic therapy as the first choice for intermediate and advanced staged HCC (16). Our previous study suggested that stereotactic therapy (i.e., a combination of systemic and locoregional therapy) could safely achieve tumour downstaging and R0 resection in HCC patients with extrahepatic metastasis (17). The overall 5-year survival rate for patients with intermediate or advanced stage HCC after downstaging and conversion followed by resection is 50%-60%, which is close to the survival rate after resection with early-stage HCC (18). Aggressive stereotactic therapy achieved simultaneous radiographic and serological responses, resulting in tumour downstaging in the present patients. Achievement of oncological unresectable conversion indicated a survival benefit after further radical resection.

Previous studies suggested that ALPPS was superior to TACE combined with portal vein embolization in the overall conversion of insufficient FLR (5). After a sufficient evaluation, we performed a pioneering ALPPS in the present patients. Although the rates of FLR hypertrophy (i.e., 4.8 mL and 3.9 mL per day) after the first stage were lower than those in patients who had not received systemic therapy (5), the second stage was finally completed within a month. This result suggested that the hypertrophic speed might be influenced by systemic therapy in addition to viral hepatitis-related fibrosis/cirrhosis (5) and intrahepatic tumour burden (19). Inflammation and cytokine release from overactivation of immune cells after treatment with ICIs may cause liver injury (20). Previous studies demonstrated that the overproduction of IFN-γ by NKT cells negatively regulated liver regeneration in HBV transgenic mice (21). Interestingly, the production of IFN-γ was decreased in NKT cells of HCC patients, and anti-PD-1 drugs could improve function of NKT cells including enhanced IFN-γ level (22). These findings may provide a potential explanation on impaired liver regeneration in HBV-related HCC patients after treatment with ICIs. However, this study had some limitations such as a lack of pathological examination on FLR and control cases. Larger samples are needed to elucidate the mechanisms for the liver regeneration influenced by ICIs.

Furthermore, it is necessary to have a useful method to predict adequate FLR hypertrophy before ALPPS (23) which was guaranteed to perform the surgery with the greatest possible safety (24). The absence of PHLF indicated a sufficient FLR for these patients. These cases demonstrate that patients with a satisfactory objective response rate according to not only the RECIST 1.1 (Case 2) but also the mRECIST guidelines (Case 1) could receive ALPPS. These patients completed conversion therapy and achieved the goal of cure. In addition, we performed first-stage ALPPS by laparoscopy rather than a conventional procedure. The use of this procedure could reduce the high rate of occurrence of procedure-related mortality and morbidity, such as bile leakage and sepsis, by diminishing invasiveness (25).

It is worth noting that in Case 1, considering the insufficiency of FLR and the toxicity of chemotherapy to the liver, the right liver lesion was selected accurately during TACE. The left liver lesion without locoregional therapy was resected in first-stage ALPPS. Interestingly, postoperative pathology revealed pCR in the large lesion of the right liver, while residual tumour cells were observed in the small lesion of the left liver. The stereotactic therapy of enhanced immunotherapy by locoregional therapy was validated in a patient with a concurrent course of disease.

FLR hypertrophy might be influenced by immunotherapy, and further researches are warranted. Nevertheless, ALPPS is feasible and safe for the treatment of selected patients with HBV-related HCC who underwent prior therapy with ICIs. ALPPS might be an alternative strategy to salvage insufficient FLR during conversion therapy.

## Ethics statement

Written informed consent was obtained from the participant/patient(s) for the publication of this case report.

## Author contributions

CN and GL collected clinical data and drafted the manuscript. JZ participated in the management of the patient. XY, YX, and HZ designed treatment plan and revised the manuscript. All authors contributed to the article and approved the submitted version.

## Glossary

**Table:**

AFP	alpha fetoprotein
ALP	alkaline phosphatase
ALPPS	associating liver partition and portal vein ligation for staged hepatectomy
ALT	alanine transaminase
AST	aspartic transaminase
BCLC	Barcelona Clinic Liver Cancer
CT	computed tomography
CTLA-4	cytotoxic T lymphocyte-associated antigen-4
DBil	direct bilirubin
FLR	future liver remnant
HBsAg	hepatitis B surface antigen
HBV	hepatitis B virus
HCC	hepatocellular carcinoma
HCV	hepatitis C virus
ICIs	immune checkpoint inhibitors
LFT	liver function test
mRECIST	modified Response Evaluation Criteria Solid Tumours
NKT	natural killer T
pCR	pathological complete response
PD-1	programmed cell death protein 1
PHLF	posthepatectomy liver failure
PR	partial response
SLV	standard liver volume
TACE	transarterial chemoembolization
TBil	total bilirubin
γ-GGT	γ gamma-glutamyl transpeptidase
